# Editorial: Endocrine consequences in children due to the COVID-19 pandemic social behavior changes

**DOI:** 10.3389/fendo.2023.1266239

**Published:** 2023-08-15

**Authors:** Guilherme Guaragna-Filho, Stefano Stagi

**Affiliations:** ^1^ Department of Pediatrics, School of Medicine, Federal University of Rio Grande do Sul, Porto Alegre, Brazil; ^2^ Department of Health Sciences, University of Florence, Firenze, Italy; ^3^ Meyer Children’s Hospital IRCCS, Florence, Italy

**Keywords:** endocrine disorders, COVID - 19, precocious puberty, T1D (type 1 diabetes), childhood obesity, thyroid disorders

Over the last 3 years, the human race has faced the greatest healthcare challenge of the 21^st^ century, a challenge made even greater, because the last time something of this magnitude occurred was over 100 years ago. To overcome the COVID 19 pandemic, the medical community was called on to use intellectual and industrial resources on a scale never seen before.

From the first cases, it was clear that the effects of COVID19 on the paediatric population would not be limited to acute respiratory conditions, but that young people’s mental health would be severely effected due to lockdowns. Endocrinological consequences, whether directly caused by the virus, as shown in some studies on DM1 (1), or multifactorial, such as the increase in cases of precocious puberty (2) soon began to appear. Evidence of collateral damage to the endocrine system continues to emerge and research into its aetiology and pathophysiology is just beginning.

This special edition focuses on the endocrinological consequences of the COVID19 pandemic in children and adolescents. A good place to start is the work of Gnocchi et al., a review of the impact of the COVID-19 pandemic on paediatric endocrine disorders, which highlights well documented phenomena such as increases in obesity as well as less researched consequences, for example the impact on vitamin D and Calcium metabolism.

A widely noted phenomenon was the increase in cases of PP. Barberi et al. present interesting data on the role of electronic devices in triggering PP during the pandemic period in Italy. Choi and Park present important data on the increase in prevalence of PP from 2016 to 2021 in the Korean population, emphasizing the prevalence in boys which increased 2-fold during the pandemic period. Chen et al. suggest that ghrelin levels play a role in the regulation of puberty based on their analysis of the increase of PP cases in Shanghai during the first year of the COVID-19 pandemic compared with the five previous years.

Alfayez et al. conducted a systematic review and metanalysis showing that diabetic ketoacidosis (DKA) risk, especially for the severe form, increased significantly during the pandemic. Abdou et al. showed that during COVID waves 1 and 2, there was an increase in the incidence of newly diagnosed cases compared with the pre-COVID period, and that patients presented with more severe DKA with a significantly higher incidence of hypokalaemia. Zubkiewicz-Kucharska et al. analysed the influence of lockdown in T1D management, finding increases in weight gain and daily insulin doses during the period.

McCowan et al. showed that overall rates of thyroid dysfunction have not altered since the beginning of the COVID-19 pandemic, however the number of patients with transient thyroid dysfunction, not requiring ongoing treatment has increased. Finally, Corica et al. describe the difficulties that the pandemic brought for the management of obesity in children and adolescents.

More studies will be needed to explain these phenomena, focusing not only on clinical presentation but also on the role of epigenetics. The covid pandemics is over, however the challenge of interpreting its effects on the paediatric population has just started for paediatric endocrinologists and healthcare professionals.

## Author contributions

GG: Writing – original draft. SS: Writing – review & editing.
